# Genome architecture: nuclear context links noncoding variants to transcriptional programs

**DOI:** 10.1038/s41392-026-02616-5

**Published:** 2026-03-12

**Authors:** Assam El-Osta

**Affiliations:** 1 Baker Heart and Diabetes Institute, Epigenetics in Human Health and Disease Program, Melbourne, Vic Australia; 2 Baker Department of Cardiometabolic Health, The University of Melbourne, Parkville, VIC Australia; 3 School of Translational Medicine, Department of Diabetes, Monash University, Melbourne, VIC Australia; 4 Department of Medicine and Therapeutics, The Chinese University of Hong Kong (CUHK), Hong Kong SAR, China

**Keywords:** Functional genomics, Molecular medicine

**Fig. 1:**
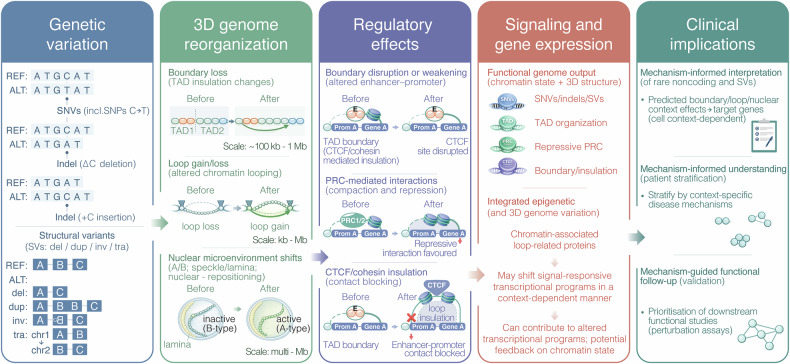
Genome architecture links genetic variation to regulatory mechanisms and clinical interpretation. *Genetic variation*. Diverse classes of genetic variation, including single-nucleotide variants (SNVs, including SNPs), small insertions and deletions (indels), and structural variants (SVs; deletions, duplications, inversions, and translocations), can perturb linear DNA sequence and genomic context, with downstream consequences for chromatin organization and nuclear architecture. *3D genome reorganization*. Genetic variants can drive genome reorganization across spatial scales, including: (i) boundary loss/altered topologically associating domain (TAD) insulation (illustrated over ~100 kb–1 Mb), (ii) chromatin loop gain or loss (kb–Mb), and (iii) nuclear microenvironment shifts, such as changes in A/B-type compartmentalization and altered association with nuclear speckles or the nuclear lamina, accompanied by nuclear repositioning (multi-Mb scale). Inactive (B-type) and active (A-type) indicate B and A compartments (repressed/lamina-associated vs active/open chromatin). “Before” and “After” schematics depict representative pre- and post-variant states. *Regulatory effects*. Architectural changes can yield multiple regulatory outcomes. Boundary disruption or weakening (disruption of a CTCF site at a TAD boundary) can rewire enhancer–promoter contacts and alter enhancer access. PRC-mediated interactions can be stabilized or redistributed, favoring compacted, repressive chromatin interactions. CTCF/cohesin insulation (contact blocking) can be gained or strengthened through loop insulation, reducing or preventing enhancer–promoter communication. In the schematic, E denotes an enhancer; Prom A denotes Promoter A associated with Gene A; and PRC1/2 denotes the Polycomb repressive complex. *Signaling and gene expression*. Integration of genetic variation (SNVs/indels/SVs) with chromatin state and 3D genome features (TAD organization, PRC repression, and boundary/insulation changes) shapes functional genome output. Altered regulatory wiring can shift signal-responsive transcriptional programs and contribute to persistent transcriptional changes, with potential feedback between signaling pathways and chromatin organization in a cell-type–dependent manner. *Clinical implications*. Mechanistic understanding of 3D genome effects supports (i) mechanism-informed interpretation of rare noncoding variants and SVs via predicted boundary/loop/nuclear-context impacts on candidate target genes, (ii) mechanism-informed patient stratification by context-specific disease mechanisms, and (iii) mechanism-guided functional follow-up, including prioritization of downstream perturbation/validation assays

In a recent study published in *Nature*,^1^ Dekker et al. present integrative 3D genome structure models that link genetic variation to nuclear microenvironmental positioning, regulatory rewiring, and downstream transcriptional programs. By connecting sequence and structural perturbations to changes in domain insulation, looping, Polycomb-associated interactions, and speckle/lamina proximity, the study advances genome architecture as a mechanistically interpretable layer for mapping variant effects onto candidate target genes in a context-dependent manner, with clear implications for variant interpretation and prioritization of functional follow-up (Fig. 1, Genetic variation).

For two decades, post-genomic biology has excelled at annotating what is encoded in the linear human genome - genes, enhancers, silencers, chromatin states, and transcription factor (TF) binding. Yet a persistent challenge in clinical genetics and cancer genomics remains: most disease-associated variants lie in noncoding regions or occur as structural variants (SVs), where “nearest gene” is often a poor guide to mechanism.^2^ The missing piece is not more 1D annotation, but a framework for how regulatory information is physically deployed in the nucleus—how chromatin folds, where loci reside relative to nuclear bodies, how these features vary across cells, and how they couple to transcription and replication (Fig. 1, 3D genome reorganization). Drawing on the NIH Common Fund 4D Nucleome (4DN) program, Dekker and colleagues provide a reference resource that connects genome architecture to function and, crucially, to variant interpretability.^1^

A major practical contribution is the insistence that the “3D genome” is not a single measurement, but a set of partially overlapping observables. The authors compare sequencing-based chromatin interaction assays that report pairwise contacts (Hi-C, Micro-C) with methods capturing multiway proximity (GAM, SPRITE), and integrate these with orthogonal approaches that locate loci relative to nuclear landmarks (TSA-seq for distances to nuclear bodies; DamID for lamina-associated contacts).^3^ This benchmarking is not academic: it defines what can be inferred from any given dataset—an essential prerequisite if 3D genome readouts are to become clinically actionable. In a concluding “user guide,” the authors highlight that compartmentalization is well captured by SPRITE and Hi-C; chromatin loops—especially structural loops—are most effectively detected by Micro-C; and enrichment-based approaches (PLAC-seq, ChIA-PET) excel at gene-expression–linked loops. With larger capture radii, SPRITE and GAM are particularly useful for detecting co-localization around nuclear bodies.

For translational readers, this method-centered framing bridges “interesting chromatin biology” to a deployable assay strategy. Patient samples are finite, heterogeneous, and noisy; clarity about what each topology readout can and cannot support is therefore pivotal. The consortium builds its integrated atlas in two widely used human cell systems—H1 human embryonic stem cells (H1-hESCs) and immortalized foreskin fibroblasts (HFFc6)—generating a multi-scale output: linear spatial annotations, extensive loop catalogs (>140,000 interactions per cell type), classifications of chromosomal domain types with subnuclear positioning, and single-cell 3D models describing the nuclear microenvironment of genes and distal contacts.^1^ Making these resources openly available through the 4DN infrastructure underscores their intent as a community reference rather than a single-study snapshot.

A particularly relevant aspect is how structural annotations are translated into mechanistic regulatory categories, effectively treating chromatin folding as an epigenetic layer shaping a signal-responsive transcriptional program (Fig. 1, Regulatory effects). The authors classify loops using chromatin-state composition at anchors, projected into low-dimensional space. Across both cell types, six loop clusters separate poised promoter-associated loops, insulator–insulator loops, and multiple transcription-linked loop types. Polycomb-group proteins (EZH2, RNF2) are enriched at anchors in the poised-promoter cluster, consistent with a model where Polycomb-associated looping contributes to compaction and repression. This observation builds on prior work linking Polycomb function to higher-order chromatin organization and boundary integrity.^2,4^ In contrast, insulator-related loops show the strongest CTCF (CCCTC-binding factor) and cohesin enrichment and often span longer genomic distances than transcription-associated loops—an architectural signature consistent with classical boundary and insulation functions.

This matters for targeted therapy because it grounds chromatin topology in druggable chromatin biology: PRC2-linked regulation, boundary integrity, and the machineries that enforce or remodel folding. While we are not yet prescribing “loop modulators,” the work strengthens the rationale that perturbing chromatin regulators (Polycomb, cohesin-associated pathways, TF networks) can produce predictable higher-order genome consequences—an angle increasingly relevant to cancer, developmental disorders, and immune dysregulation^5^ (Fig. 1, Signaling and gene expression).

A second axis of “epigenetic control” is nuclear organization: proximity to speckles, lamina, nucleoli, and radial positioning as determinants of transcriptional output. Using genome structure models, the authors report that ~90% of the most highly expressed genes show high-to-medium association frequencies with nuclear speckles in the studied fibroblast context, illustrating a broader link between nuclear positioning and transcriptional activity. They also note informative heterogeneity: genes silent in pluripotent cells can exhibit bimodal nuclear location distributions, consistent with cell-to-cell structural variability or subpopulations in distinct epigenetic states. From a signaling perspective, this provides a physical substrate for “transcriptional competence,” extending earlier work demonstrating that nuclear compartmentalization constrains gene regulation.

The study’s most translationally catalytic section is the demonstration that genome folding can be predicted from sequence using deep learning, enabling in silico testing of variant effects. Using Micro-C data and the Akita architecture, the authors predict contact-map changes from sequence perturbations, including a 345-bp deletion at the TAL1 locus that removes a CTCF site at a TAD boundary and increases predicted contacts between TAL1 and adjacent regions. They extend this approach through motif perturbations at cell-type-specific boundaries, supporting a context-dependent “boundary grammar” in which TFs contribute to architectural specificity. Importantly, the models are framed not only as predictors but as hypothesis generators, offering a tractable path from contact maps to mechanism. The authors also note a practical limitation—deep learning may underestimate true variant effects—helping calibrate expectations for clinical translation.

For medical genetics, the conceptual shift is equally important: variant interpretation can move from proximity heuristics to causal models of regulatory rewiring (Fig. 1, Clinical implications). In rare disease, a noncoding variant may be prioritized because it is predicted to weaken insulation at a boundary, altering enhancer access to a dosage-sensitive developmental gene. In cancer, an SV may be interpreted as enhancer hijacking because it reconfigures TAD structure and nuclear neighborhood, reshaping contact probabilities and transcriptional outputs. In both settings, “4D nucleome reasoning” offers a mechanistic language that clinicians and molecular tumor boards can operationalize.

Signal transduction is often narrated as a straight line - from ligand- to- receptor, kinase cascades to TFs, and finally, a gene program. This work argues that the last step, gene-program execution, cannot be understood without the physical genome. Enhancers do not regulate “targets” in the abstract; they regulate what they can contact within a nuclear landscape that is organized, heterogeneous, and—crucially—partly predictable from sequence. By pairing a rigorously benchmarked atlas with a computational bridge from sequence to folding, Dekker et al. provide a practical blueprint for turning chromatin topology into a clinically interpretable layer of regulation rather than a descriptive curiosity.
